# Potentially inappropriate testing for vitamin D deficiency: a cross-sectional study in Switzerland

**DOI:** 10.1186/s12913-020-05956-2

**Published:** 2020-11-27

**Authors:** Stefan Essig, Christoph Merlo, Oliver Reich, Maria Trottmann

**Affiliations:** 1 Institute of Primary and Community Care, Schwanenplatz 7, 6004 Luzern, Switzerland; 2 santé24, Palmstrasse 26b, 8401 Winterthur, Switzerland; 3 SWICA Health Services Research, Römerstrasse 38, 8401 Winterthur, Switzerland

**Keywords:** Vitamin D, Vitamin D deficiency, Diagnosis, Medical overuse, Underuse, Standards, Evidence-based Medicine

## Abstract

**Background:**

There is consensus that vitamin D supplementation is often indicated but population-based screening by laboratory testing for vitamin D deficiency is inadequate. Testing should be restricted to people at high risk of severe deficiency. This study describes the current lab testing for vitamin D deficiency in the adult population of Switzerland.

**Methods:**

We assessed Swiss health insurance data (SWICA) for incidence of lab testing for vitamin D levels, comparing the years 2015 and 2018. Claims were analyzed for associations between lab testing and age, sex, medical indications, insurance status and geographic location in multivariable regression analyses. We also estimated the costs of vitamin D testing.

**Results:**

Data from 200,043 and 200,046 persons for 2015 and 2018, respectively, were analyzed. Vitamin D level was tested in 14% of the sample population in 2015 and 20% in 2018. Testing increased by 69% for individuals aged 26–30. Testing was associated with being middle-aged to young senior citizens, female, medical indications (pregnancy, renal disease, osteoporosis, hyperparathyroidism, HIV, glucocorticoid intake), more chronic conditions, having a mandatory insurance with a low deductible, additional insurance coverage, and living in urban areas. We estimate that the total laboratory cost to mandatory insurance was about 90 million Swiss francs in 2018.

**Conclusions:**

Despite recommendations for routine vitamin D supplementation, vitamin D testing of low risk individuals is common and increasing in Switzerland.

## Background

Low vitamin D levels are common [1, 2]. However, there is no clear figure for the prevalence of vitamin D deficiency, as the cut-off between normal and low values varies and laboratory assays are not standardized [3]. Low serum levels of vitamin D (i.e., 25-hydroxyvitamin D) are a causal factor for fractures [4] and are associated with many other health issues such as heart disease, type 2 diabetes, and dementia [5–7]. According to Swiss guidelines, patients with (a high risk of) bone diseases, such as osteoporosis or hyperparathyroidism; older, obese, or dark-skinned people; pregnant and breastfeeding women with risk factors; patients with chronic renal disease, liver failure, malabsorption, granulomatous diseases; and patients who are taking certain drugs (anticonvulsants, glucocorticoids, antiretroviral, and others) should be tested [8]. These conditions justify a blood test, as the risk of seriously low levels is high [9, 10]. Testing allows to identify and meet the need for increased doses of vitamin D of up to 2000 IU per day. The dosing regimens are under scientific scrutiny [11, 12], but should certainly be carefully selected based on the underlying condition and the norms of the tolerable upper intake level of vitamin D [13].

However, tests for vitamin D deficiency are not always helpful [9]. Many people have insufficient levels (< 50 nmol/l; approximately 59% of patients in Swiss primary care [1]), but few have severely deficient levels (< 25 nmol/l; 12% of patients). Guidelines say that testing is therefore often unnecessary, as physicians can generally advise patients to get enough vitamin D, regardless of test results [14]. Discussions should include the intake of vitamin D supplements. Over-the-counter vitamin D has been suggested as appropriate routine prevention in the general population of Switzerland and many other countries; the recommended doses vary mostly between 400 and 800 IU per day [8, 11, 15]. Other discussion items include the role of finding the right balance between exposure to and protection from the sun, and behavioral changes such as smoking cessation and increased physical activity [16]. Even if patients are suffering from chronic conditions such as diabetes and heart disease, lab values are not necessarily helpful [17]. Since population-based screening is questionable, the total cost of vitamin D tests have been identified as an unnecessary economic burden in many health care systems [18]. For example, “Do not do” lists of “Choosing Wisely” campaigns now include “Do not sperform population-based screening for vitamin D deficiency” in the US [19] and “Do not routinely measure vitamin D in low-risk adults” in Canada [20]. Potentially inappropriate testing is present if people get tested who do not belong to risk groups specified in the guidelines–or people do not get tested but belong to risk groups. It is therefore insightful to analyze whether testing is associated with factors that are not mentioned in the guidelines–or not associated with factors that are mentioned.

This study aims to describe lab testing for vitamin D levels in the adult population of a large Swiss insurance company. We compared the proportion of the population that received testing for vitamin D between 2015 and 2018 and determined the association between characteristics of the population and testing. We also estimated the total costs of vitamin D testing.

## Methods

In Switzerland, health insurance is compulsory and can be obtained from over 50 insurance funds. Insurance covers all medical services provided or prescribed by physicians, including testing for vitamin D. Health plans vary by deductibles and may include managed-care type restrictions such as gatekeeping by a general practitioner, gatekeeping by a telemedical provider, or health maintenance organization. People can take out supplemental coverage for hospital care. Supplemental coverage pays for additional services such as free choice of hospital and surgeon and more luxurious rooms, and is strongly associated with having higher socioeconomic status [21].

A major Swiss health insurance fund (SWICA, Winterthur, Switzerland) provided anonymized claims data for the years 2015 and 2018. Out of all 800,000 insured persons, the company drew a purposive sample of approximately 200,000 people for both years. The sample was stratified based on the Swiss population in terms of age and geographic distribution (see Supplementary File S1 for a more detailed description of the stratification procedure) [22]. Patient age of at least 18 years was the only inclusion criterion. We defined the time at risk as the sum of the individual time spans of being insured in patient-years to account for those persons acquiring or dropping insurance coverage during the year.

All variables came from claims data in either 2015 or 2018. Information on gender, age, geographic region, and urbanity level (according to the Federal Statistical Office [23]) were readily available. The age variable was categorized in 20-year groups (19–40, 41–60, 61–80, 81+ years) for brevity; in addition, we repeated the analyses with groups of 5 years, and age as a continuous variable. We used variables on the chosen deductible class (300, 500, 1000, 1500, 2000, or 2500 CHF) and health plan from mandatory insurance, and supplemental coverage for hospital care. Swiss-adapted pharmaceutical cost groups (PCG) were used to determine a variable on chronic conditions (zero, one, two, and at least three conditions) [24, 25].

Codes of services from the Swiss classification of laboratory tests (Analysis list; AL), anatomical therapeutic chemical groups (ATC), diagnosis related groups (DRG), and outpatient medical services (TARMED) were used to determine variables for blood tests, supplementation and health conditions. Blood tests for vitamin D can be directly identified in claims data (AL position 1006.00). Medications reimbursed by mandatory insurance and medical claims were used to indicate vitamin D supplementation (ATC A11CB, A11CC, A12AX), and conditions that are indications for vitamin D testing according to Swiss guidelines [8]: renal disease (3+ packages ATC B03XA01, B03XA02, B03XA03, V03AE01, V03AE02, V03AE03, V03AE04), osteoporosis (3+ packages ATC M05BA, M05BB, H05BA,G03XC, M05BX04, H05AA02, M05BX53; or DRG I69; or TARMED 39.2140, 39.2150, 39.2160), epilepsy (3+ packages ATC N03AA, N03AB, N03AF), hyperparathyroidism (3+ packages ATC H05BX01, H05BX02, H05BX04, V03AE02, A02AB01, V03AE07), HIV (3+ packages ATC J05AR) and intake of corticosteroids (3+ packages ATC H02). Pregnancy was identified based on claims related to maternity that are exempt from copayments.

We assigned the citizens to groups tested and non-tested for vitamin D. We determined the proportion of the population in 2015 and 2018 that received testing divided into age groups with a range of 5 years. We report percentages, risk ratios, and 95% confidence intervals. Furthermore, we conducted a multivariable logistic regression analysis to assess associations between the previously mentioned variables and vitamin D testing. We report odds ratios and 95% confidence intervals. Furthermore, The purposive sample allowed us to extrapolate the costs of lab tests to all insured people in Switzerland. Claims data were selected and prepared using Oracle SQL and statistical analyses were performed with R version 3.4.3.

## Results

We included data from 200,043 persons, providing 195,281 person-years in 2015, and from 200,046 persons, providing 195,458 person-years in 2018. Population characteristics for 2018 (Table 1) were very similar to those for 2015 (Supplementary Table S2).

**Table 1:** Characteristics of population in 2018, stratified by lab testing for vitamin D level

		Population not tested for vitamin D level (*N* = 157,073)	Population tested for vitamin D level (*N* = 38,384)
Variable		Percentage (95% confidence interval)	Percentage (95% confidence interval)
Gender	Male	50.9 (50.7–51.2)	32.4 (32.0–32.9)
Age group in years	19–40	37.6 (37.3–37.8)	22.6 (22.1–23.0)
	41–60	36.6 (36.3–36.8)	35.0 (34.5–35.5)
	61–80	20.8 (20.6–21.0)	33.5 (33.0–33.9)
	81+	5.0 (4.9–5.1)	9.0 (8.7–9.3)
Deductible class in CHF	300	39.4 (39.2–39.6)	59.7 (59.2–60.2)
	500	12.4 (12.3–12.6)	17.0 (16.6–17.4)
	1000	5.6 (5.4–5.7)	4.3 (4.1–4.5)
	1500	10.1 (10.0–10.3)	5.5 (5.3–5.7)
	2000	5.4 (5.2–5.5)	2.4 (2.3–2.6)
	2500	27.1 (26.9–27.3)	11.0 (10.7–11.3)
Health plan	Unrestricted access	14.3 (14.1–14.5)	22.6 (22.2–23.0)
	Gatekeeping by GP	25.4 (25.2–25.6)	26.1 (25.7–26.6)
	Gatekeeping by telemedical provider	28.7 (28.5–29)	26.5 (26.1–27.0)
	HMO	31.6 (31.3–31.8)	24.7 (24.3–25.2)
Supplementary insurance coverage	Yes	24.2 (24.0–24.4)	29.6 (29.1–30.0)
Geographic region	Eastern	14.6 (14.4–14.8)	11.9 (11.5–12.2)
	Central	9.8 (9.6–9.9)	9.0 (8.7–9.3)
	Mittelland	22.3 (22.1–22.5)	21.6 (21.2–22.0)
	Northwest	13.7 (13.5–13.9)	14.3 (13.9–14.6)
	Western	21.6 (21.4–21.8)	25.4 (25.0–25.9)
	Zurich	18.0 (17.8–18.2)	17.8 (17.4–18.2)
Urbanity levels^a^	Rural	10.5 (10.3–10.6)	8.5 (8.2–8.8)
	Peri-urban	22.1 (21.9–22.3)	19.3 (18.9–19.7)
	Urban small	8.8 (8.7–9.0)	8.4 (8.1–8.6)
	Urban midsize	27.8 (27.6–28.1)	27.1 (26.7–27.6)
	Urban large	30.8 (30.5–31.0)	36.7 (36.2–37.2)
Pregnancy	Yes	2.4 (2.3–2.4)	3.2 (3.1–3.4)
Chronic morbidities^b^	0	74.2 (74.0–74.4)	47.7 (47.2–48.2)
	1	13.6 (13.4–13.7)	22.5 (22.1–23.0)
	2	7.2 (7.1–7.3)	15.4 (15.0–15.7)
	≥3	5.0 (4.9–5.1)	14.5 (14.1–14.8)
Renal disease^b^	Yes	0.03 (0.02–0.04)	0.3 (0.2–0.3)
Osteoporosis^b^	Yes	1.1 (1.1–1.2)	6.8 (6.5–7.0)
Epilepsy^b^	Yes	0.2 (0.2–0.2)	0.4 (0.3–0.4)
Hyperparathyroidism^b^	Yes	0.01 (0.01–0.02)	0.2 (0.1–0.2)
HIV^b^	Yes	0.1 (0.1–0.2)	0.4 (0.4–0.5)
Glucocorticoids^b^	Yes	1.7 (1.7–1.8)	5.1 (4.9–5.3)
Vitamin D supplementation^b^	Yes	4.0 (3.9–4.1)	23.6 (23.2–24.0)

*CHF* Swiss francs, *GP* General practitioner, *HMO* Health Maintenance Organization, *HIV* Human Immunodeficiency Virus

^a^based on definitions of Federal Statistical Office

^b^based on pharmaceutical claims

**Fig. 1:**
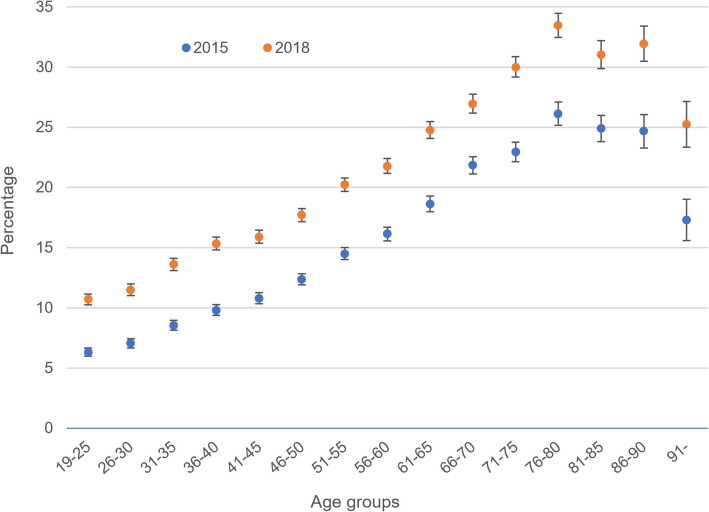
Percentage of population that received testing for vitamin D in 2015 and 2018, stratified by age group (95% confidence intervals; unadjusted analysis)

In our study population, vitamin D level was tested in 14% of the study population in 2015 and 20% in 2018. Incidence increased in all age groups from the 2015 to the 2018 sample. The most pronounced increase was observed among individuals aged 26–30, where testing incidence rose from 7 to 12%, reflecting an increase of 69%. All differences are statistically significant (Fig. 1). The underlying numbers of the figure are provided in Supplementary Table S3.

In 2018, an age of 41–60 and 61–80 years and female gender were associated with being tested for vitamin D levels. Pregnancy, a higher number of chronic morbidities, renal disease, osteoporosis, hyperparathyroidism, HIV, intake of glucocorticoids, and vitamin D supplementation were also positively associated with vitamin D testing. Furthermore, there were positive associations with additional insurance coverage, living in urban areas, and a low deductible class. An age of 81+ years and HMO model in mandatory insurance was associated with not being tested. These associations were significant on a 95% confidence level (Table 2). The additional analyses offer more detailed insights; with age groups of 5 years, the probability of being tested increased with age until 55 years, and then decreased again (Supplementary Table S4); to include age as continuous variable and account for this nonlinearity, we specified age and age squared as regressors (Supplementary Table S5); the marginal effects are positive in young age groups, and negative in high age groups (Supplementary Table S6).

**Table 2:** Association of characteristics and testing for vitamin D level in 2018 (multivariable regression analysis)

Variable		Odds Ratio (95% confidence interval)
Gender	Female	1
	Male	0.56 (0.55–0.58)
Age group in years	19–40	1
	41–60	1.30 (1.25–1.34)
	61–80	1.20 (1.15–1.24)
	81+	0.75 (0.71–0.80)
Deductible class in CHF	300	1
	500	0.95 (0.92–0.98)
	1000	0.71 (0.67–0.76)
	1500	0.57 (0.55–0.60)
	2000	0.51 (0.48–0.55)
	2500	0.46 (0.44–0.48)
Health plan	Unrestricted access	1
	Gatekeeping by GP	1.02 (0.98–1.06)
	Gatekeeping by telemedical provider	1.05 (1.01–1.09)
	HMO	0.86 (0.83–0.89)
Supplementary insurance coverage	No	1
	Yes	1.21 (1.18–1.24)
Geographic region	Eastern	1
	Central	1.04 (0.98–1.09)
	Mittelland	1.06 (1.02–1.11)
	Northwestern	1.01 (0.96–1.07)
	Western	1.13 (1.08–1.19)
	Zurich	1.00 (0.96–1.06)
Urbanity levels^a^	Rural	1
	Peri-urban	1.11 (1.05–1.16)
	Urban small	1.12 (1.05–1.18)
	Urban midsize	1.22 (1.16–1.28)
	Urban large	1.39 (1.32–1.46)
Pregnancy	No	1
	Yes	1.88 (1.75–2.02)
Chronic morbdities^b^	0	1
	1	1.83 (1.77–1.90)
	2	2.09 (2.01–2.18)
	≥3	2.37 (2.26–2.48)
Renal disease^b^	No	1
	Yes	1.87 (1.27–2.75)
Osteoporosis^b^	No	1
	Yes	2.17 (2.02–2.32)
Epilepsy^b^	No	1
	Yes	0.92 (0.75–1.14)
Hyperparathyroidism^b^	No	1
	Yes	2.78 (1.64–4.72)
HIV^b^	No	1
	Yes	1.66 (1.33–2.06)
Glucocorticoids^b^	No	1
	Yes	1.43 (1.34–1.53)
Vitamin D supplementation^b^	No	1
	Yes	3.96 (3.81–4.11)

*CHF* Swiss francs, *GP* General practitioner model, *HMO* Health Maintenance Organization, *HIV* Human Immunodeficiency Virus

^a^based on definitions of Federal Statistical Office

^b^based on pharmaceutical claims

In 2015, the associated variables were the same (Supplementary Table S7). In terms of effect size, the associations of the age groups 41–60 and 61–80 with being tested for vitamin D levels were more pronounced. The negative association of age 81+ was less pronounced.

The total cost of blood tests for vitamin D deficiency amounted to CHF 2.6 million (1 CHF is roughly 0.95 EUR) in the sample in 2018, compared to CHF 1.8 million in 2015. Extrapolating this cost to all insured people in Switzerland, we estimate that total cost to mandatory insurance was about CHF 90 million in 2018, compared to CHF 61 million in 2015.

## Discussion

Vitamin D level was tested in 14% of the study population in 2015 and 20% in 2018. Between 2015 and 2018, incidence increased in all age groups, with the greatest increase observed for young individuals. We found significant associations of variables with vitamin D testing that are no indications for testing according to Swiss guidelines. The associated variables include having a low deductible class in mandatory insurance, possession of additional insurance coverage (which is related to higher socioeconomic status), and residence in urban areas. We estimate that total laboratory cost to mandatory insurance in Switzerland was about 90.4 million CHF in 2018.

The strengths of this study include the sample size and nationwide sampling frame. Another important aspect is the short observation period of 3 years, limiting the risk that unmeasured parameters might have changed, i.e., the availability and marketing of tests, population preferences and regulatory settings. Limitations are the retrospective and observational design, which excludes the possibility of drawing causal inference. Using a single health insurance among the multiple insurers in Switzerland is another limitation, but the sample size and stratification in terms of age and geographic location counteract this potential source of bias. Furthermore, we cannot present a precise and complete picture of diseases and treatments that are rightly linked to vitamin D testing, because we cannot infer all morbidities from claims data. The included morbidities are relevant and of rather high prevalence. The inclusion of rarer diseases and genetic predispositions in the analysis would most probably change the results insignificantly. Medication that was paid out-of-pocket and not submitted to the insurance could not be accounted for. Our data did not include personal factors that are linked to vitamin D deficiency, such as skin color or overweight. Last, we did not know if a vitamin D deficiency was present or not. These sources of residual confounding can lead to an underestimation or overestimation of the appropriateness of testing. If underestimated, we missed some of the inappropriate testing–too much or too little–which will therefore not be tackled. These inefficiencies in health care delivery can become more and more costly and a risk for patients’ health. If overestimated, i.e., we describe more inappropriate testing than really exists, resources might be waisted to improve allocation, with most burden on research and policy developers.

The overall results indicate potentially inappropriate testing that is present alongside valid indications of medical conditions according to Swiss guidelines. For elderly people, Vitamin D testing may be interpreted as insufficient. The oldest old citizens have a high risk of severely low levels of vitamin D but were less often tested than young senior citizens. This finding is comparable to results from other medical fields, suggesting that advanced age can be associated with lower rates of diagnostic testing [26]. On the other hand, the reported testing frequency can be viewed as too high from the perspective that vitamin D testing should be restricted to persons at high risk of low levels. Considering the particularly large increase of testing among young people within 3 years, and the association of testing with socioeconomic variables that might indicate supply-sensitive care, it seems indeed possible that too many tests are performed. The main underlying mechanism might be a general trend for overuse of medical services and tests in recent years, with vitamin D testing proving no exception [27, 28]. Some authors have declared a “pandemic” of vitamin D deficiency [2, 6] which may be a cause of our results, as awareness and demand for tests increases [18].

The findings of the current article are in line with those of previous publications. Numerous studies from other countries have shown similarly high rates of vitamin D testing [28]. A recent scoping review identified thirteen studies from seven countries that were published between 2006 and 2015 and consistently reported an increase in vitamin D testing [29]. For example, vitamin D testing increased by 59% per year between 2000 and 2010 in Australia [30]. Additionally, authors from the UK found that the annual number of tests sharply increased between 2008 and 2016, and that 70–78% of tests were potentially inappropriate [31]. Compared to our article, these studies include prior years and a longer observation period. Other studies have gone one step further in trying to improve the allocation of vitamin D tests in practice. An Australian study showed that a strategy to tackle the number of tests does not necessarily correlate with more appropriate testing [32]. Another study focused on improving vitamin D supplementation in aged care without measuring vitamin D levels, but care facilities still identified physicians’ beliefs and attitudes related to blood tests as a barrier to routine supplementation [33].

This study has several implications. From an epidemiological perspective, clinicians should better consider the pre-test chance of very low values of vitamin D. If the risk of severe vitamin D deficiency is high, i.e., patients belong to the pre-specified risk groups, the test is appropriate. If the risk is low–especially in generally young, healthy people–the test can mostly be avoided, and preventive measures and possibly supplements may be easier options. Following these suggestions would reduce the risk of inappropriate testing. Guidelines for routine supplementation without testing will have to be clear in order to avoid overdosing. It is unclear if the Choosing Wisely campaign in Switzerland (“Smarter Medicine”) will phrase a “Do not do” as their colleagues from Canada and the US. Rising costs of approximately 90 million Swiss francs to mandatory insurance seem excessive for a single diagnostic test in the adult population. As a frame of reference, the total cost of laboratory analyses was approximately 1.6 billion Swiss francs (including children) [34]. Active quality improvement circles and further dissemination of the current results are needed to inform physicians and patients.

Future research and policy should determine the drivers of inappropriate testing and elucidate strategies on how we can improve the allocation of vitamin D testing to patients, i.e., eliminate wasteful use of the test. Developing and evaluating clinical decision support tools might be worthwhile; it will be important to see how the individual decision-making affects testing. Furthermore, prospective studies with pre-defined criteria and more detailed information on morbidities that are indications for vitamin D testing would complement our claims-based study. The potentially causal connection between vitamin D testing and variables remained unanswered by the current study. The cost estimation could be continued including data of physician visits and medication. The fields of pediatrics, diagnostic cut-offs for vitamin D deficiency in blood tests, and recommendations for vitamin D supplementation will also be important but are not part of the current study. Overall, we should better understand how vitamin D deficiency is defined, measured, and treated.

## Conclusions

Despite recommendations for routine vitamin D supplementation, vitamin D testing of low risk individuals is common and increasing in Switzerland.

## Supplementary Information

**Additional file 1: File S1**. Prevalences of regions and age groups in the Swiss population and the stratified sample.

**Additional file 2: Table S2**. Characteristics of population in 2015, stratified by lab testing for vitamin D level.

**Additional file 3: Table S3**. Percentage of population that received testing for vitamin D in 2015 and 2018, stratified by age group, and ratio between 2015 and 2018.

**Additional file 4: Table S4**. Alternative regression results: Age in 5-year groups.

**Additional file 5: Table S5**. Alternative regression results: Age as a continuous variable.

**Additional file 6: Table S6**. Alternative regression results: Estimated average marginal effects of 10-year increase in age.

**Additional file 7: Table S7**. Association of characteristics and testing for vitamin D level in 2015 (multivariable regression analysis).

## Data Availability

The datasets generated and analysed during the current study are not publicly available due to data protection considerations but are available from MT on reasonable request and signature of a data protection contract. SWICA claims data may under no circumstances be combined with other data sources that may allow identification of individuals in the sample.

## Abbreviations

AL: Analysis list
ATC: Anatomical therapeutic chemical groups
CHF: Swiss francs
DRG: Diagnosis related groups
EUR: Euro
GP: General practitioner model
HIV: Human immunodeficiency virus
HMO: Health maintenance organization
PCG: Pharmaceutical cost groups
TARMED: Tariff system for outpatient medical services
