# Supplementary material for: Potentially inappropriate testing for vitamin D deficiency: a cross-sectional study in Switzerland
Source: BMC Health Serv Res. 2020 Nov 27;20:1097. doi: 10.1186/s12913-020-05956-2 (PMC7694269; doi:10.1186/s12913-020-05956-2)
Supplement: Supplementary file 1 — Additional file 1: File S1. Prevalences of regions and age groups in the Swiss population and the stratified sample. [file 12913_2020_5956_MOESM1_ESM.docx]

**File S1 Online Appendix. Prevalences of regions and age groups in the Swiss population and the stratified sample**

SWICA is a major Swiss health insurer enrolling about 800'000 individuals or 10% of the Swiss population. For historical reasons, SWICA enrolls a greater share of the population in the eastern part of Switzerland and in the Zurich area. In order to make the study representative for the Swiss population, we drew a stratified the sample according to age and geographical region. Age was grouped into five year intervals, because this grouping is also used to calculate Swiss risk adjustment. As for the geographical distribution, the Swiss cantons were grouped into six groups according to a typology also used by the Federal Statistical Office. Official data from the Swiss risk adjustment fund was used in order to calculate the distribution of these groups in the Swiss population (Statistik Risikoausgleich: https://www.kvg.org/de/statistik-_content---1--1052.html). In order to obtain a sample with the same distribution, SWICA enrollees were drawn without replacement. The group with the minimum number of individuals relative to the Swiss population determined the possible size of the total sample.

|  |  | Age group | Prevalence in the Swiss population | Sample prevalence |
| --- | --- | --- | --- | --- |
| **Western and Ticino** | | **all** | **22.66%** | **22.39%** |
| Western and Ticino | | 19-25 | 2.16% | 2.06% |
| Western and Ticino | | 26-30 | 1.88% | 1.82% |
| Western and Ticino | | 31-35 | 1.92% | 1.89% |
| Western and Ticino | | 36-40 | 1.97% | 1.94% |
| Western and Ticino | | 41-45 | 2.02% | 2.00% |
| Western and Ticino | | 46-50 | 2.13% | 2.14% |
| Western and Ticino | | 51-55 | 2.21% | 2.21% |
| Western and Ticino | | 56-60 | 1.89% | 1.89% |
| Western and Ticino | | 61-65 | 1.52% | 1.52% |
| Western and Ticino | | 66-70 | 1.35% | 1.34% |
| Western and Ticino | | 71-75 | 1.28% | 1.28% |
| Western and Ticino | | 76-80 | 0.95% | 0.95% |
| Western and Ticino | | 81-85 | 0.69% | 0.69% |
| Western and Ticino | | 86-90 | 0.44% | 0.43% |
| Western and Ticino | | 91- | 0.25% | 0.23% |
| **Mittelland** |  | **all** | **22.20%** | **22.17%** |
| Mittelland |  | 19-25 | 2.09% | 2.03% |
| Mittelland |  | 26-30 | 1.78% | 1.77% |
| Mittelland |  | 31-35 | 1.81% | 1.81% |
| Mittelland |  | 36-40 | 1.81% | 1.82% |
| Mittelland |  | 41-45 | 1.78% | 1.79% |
| Mittelland |  | 46-50 | 2.01% | 2.03% |
| Mittelland |  | 51-55 | 2.14% | 2.17% |
| Mittelland |  | 56-60 | 1.94% | 1.96% |
| Mittelland |  | 61-65 | 1.65% | 1.66% |
| Mittelland |  | 66-70 | 1.47% | 1.49% |
| Mittelland |  | 71-75 | 1.33% | 1.34% |
| Mittelland |  | 76-80 | 0.96% | 0.96% |
| Mittelland |  | 81-85 | 0.71% | 0.70% |
| Mittelland |  | 86-90 | 0.46% | 0.44% |
| Mittelland |  | 91- | 0.24% | 0.21% |
| **Northwestern** |  | **all** | **13.74%** | **13.80%** |
| Northwestern |  | 19-25 | 1.21% | 1.19% |
| Northwestern |  | 26-30 | 1.10% | 1.10% |
| Northwestern |  | 31-35 | 1.18% | 1.18% |
| Northwestern |  | 36-40 | 1.19% | 1.19% |
| Northwestern |  | 41-45 | 1.13% | 1.14% |
| Northwestern |  | 46-50 | 1.25% | 1.26% |
| Northwestern |  | 51-55 | 1.36% | 1.38% |
| Northwestern |  | 56-60 | 1.21% | 1.23% |
| Northwestern |  | 61-65 | 1.02% | 1.03% |
| Northwestern |  | 66-70 | 0.87% | 0.88% |
| Northwestern |  | 71-75 | 0.79% | 0.80% |
| Northwestern |  | 76-80 | 0.60% | 0.60% |
| Northwestern |  | 81-85 | 0.44% | 0.44% |
| Northwestern |  | 86-90 | 0.28% | 0.27% |
| Northwestern |  | 91- | 0.14% | 0.12% |
| **Eastern** |  | **all** | **13.99%** | **14.08%** |
| Eastern |  | 19-25 | 1.37% | 1.34% |
| Eastern |  | 26-30 | 1.18% | 1.18% |
| Eastern |  | 31-35 | 1.18% | 1.18% |
| Eastern |  | 36-40 | 1.14% | 1.15% |
| Eastern |  | 41-45 | 1.09% | 1.10% |
| Eastern |  | 46-50 | 1.24% | 1.26% |
| Eastern |  | 51-55 | 1.38% | 1.40% |
| Eastern |  | 56-60 | 1.26% | 1.28% |
| Eastern |  | 61-65 | 1.06% | 1.07% |
| Eastern |  | 66-70 | 0.91% | 0.92% |
| Eastern |  | 71-75 | 0.79% | 0.81% |
| Eastern |  | 76-80 | 0.59% | 0.60% |
| Eastern |  | 81-85 | 0.42% | 0.43% |
| Eastern |  | 86-90 | 0.26% | 0.25% |
| Eastern |  | 91- | 0.13% | 0.12% |
| **Central** |  | **all** | **9.59%** | **9.62%** |
| Central |  | 19-25 | 0.91% | 0.89% |
| Central |  | 26-30 | 0.79% | 0.79% |
| Central |  | 31-35 | 0.82% | 0.82% |
| Central |  | 36-40 | 0.82% | 0.82% |
| Central |  | 41-45 | 0.79% | 0.81% |
| Central |  | 46-50 | 0.90% | 0.91% |
| Central |  | 51-55 | 0.98% | 0.99% |
| Central |  | 56-60 | 0.86% | 0.87% |
| Central |  | 61-65 | 0.70% | 0.71% |
| Central |  | 66-70 | 0.59% | 0.60% |
| Central |  | 71-75 | 0.51% | 0.52% |
| Central |  | 76-80 | 0.39% | 0.39% |
| Central |  | 81-85 | 0.28% | 0.28% |
| Central |  | 86-90 | 0.17% | 0.17% |
| Central |  | 91- | 0.08% | 0.07% |
| Zurich |  | all | 17.82% | 17.94% |
| Zurich |  | 19-25 | 1.49% | 1.47% |
| Zurich |  | 26-30 | 1.56% | 1.56% |
| Zurich |  | 31-35 | 1.79% | 1.80% |
| Zurich |  | 36-40 | 1.79% | 1.81% |
| Zurich |  | 41-45 | 1.64% | 1.65% |
| Zurich |  | 46-50 | 1.66% | 1.69% |
| Zurich |  | 51-55 | 1.69% | 1.71% |
| Zurich |  | 56-60 | 1.41% | 1.43% |
| Zurich |  | 61-65 | 1.14% | 1.16% |
| Zurich |  | 66-70 | 1.00% | 1.01% |
| Zurich |  | 71-75 | 0.93% | 0.94% |
| Zurich |  | 76-80 | 0.71% | 0.72% |
| Zurich |  | 81-85 | 0.52% | 0.52% |
| Zurich |  | 86-90 | 0.32% | 0.31% |
| Zurich |  | 91- | 0.16% | 0.15% |
