# Supplementary material for: Potentially inappropriate testing for vitamin D deficiency: a cross-sectional study in Switzerland
Source: BMC Health Serv Res. 2020 Nov 27;20:1097. doi: 10.1186/s12913-020-05956-2 (PMC7694269; doi:10.1186/s12913-020-05956-2)
Supplement: Supplementary file 2 — Additional file 2: Table S2. Characteristics of population in 2015, stratified by lab testing for vitamin D level. [file 12913_2020_5956_MOESM2_ESM.docx]

**Table S2 Online Appendix. Characteristics of population in 2015, stratified by lab testing for vitamin D level**

|  |  | Population not tested for vitamin D level (N=167,979) | Population tested for vitamin D level (N=27,302) |
| --- | --- | --- | --- |
| Variable |  | Percentage  (95% confidence interval) | Percentage  (95% confidence interval) |
| Gender | Male | 50.2 (50.0–50.4) | 30.4 (29.9–31.0) |
| Age group in years | 19–40 | 37.3 (37.1–37.5) | 19.7 (19.2–20.1) |
|  | 41–60 | 37.1 (36.9–37.3) | 35.3 (34.7–35.8) |
|  | 61–80 | 20.6 (20.4–20.8) | 35.5 (35.0–36.1) |
|  | 81+ | 5.0 (4.9–5.1) | 9.5 (9.2–9.9) |
| Deductible class in CHF | 300 | 39.3 (39.1–39.6) | 58.9 (58.3–59.5) |
|  | 500 | 14.6 (14.4–14.7) | 19.4 (18.9–19.8) |
|  | 1000 | 7.1 (7.0–7.2) | 4.5 (4.7–5.3) |
|  | 1500 | 13.8 (13.7–14.0) | 7.2 (6.9–7.5) |
|  | 2000 | 8.5 (8.3–8.6) | 3.1 (2.9–3.3) |
|  | 2500 | 16.7 (16.5–16.9) | 6.4 (6.1–6.7) |
| Health plan | Unrestricted access | 17.2 (17.0–17.4) | 27.7 (27.2–28.2) |
|  | Gatekeeping by GP | 28.1 (27.9–28.3) | 28.1 (27.6–28.7) |
|  | Gatekeeping by telemedical provider | 30.8 (30.6–31.0) | 25.5 (25.0–26.0) |
|  | HMO | 23.9 (23.7–24.1) | 18.7 (18.2–19.1) |
| Supplementary insurance coverage | Yes | 26.3 (26.1–26.5) | 33.8 (33.3–34.4) |
| Geographic region | Eastern | 14.7 (14.6–14.9) | 10.8 (10.4–11.2) |
|  | Central | 9.7 (9.6–9.9) | 8.6 (8.3–9.0) |
|  | Mittelland | 22.3 (22.2–22.5) | 22.1 (21.6–22.6) |
|  | Northwest | 13.6 (13.5–13.8) | 14.5 (14.1–14.9) |
|  | Western | 21.7 (21.6–21.9) | 26.2 (25.7–26.7) |
|  | Zurich | 17.8 (17.6–18.0) | 17.8 (17.4–18.3) |
| Urbanity levels^a^ | Rural | 10.6 (10.4–10.7) | 8.1 (7.8–8.4) |
|  | Peri-urban | 22.0 (21.8–22.2) | 18.9 (18.4–19.3) |
|  | Urban small | 8.9 (8.8–9.1) | 7.7 (7.4–8.0) |
|  | Urban midsize | 29.2 (29.0–29.4) | 26.4 (25.9–27.0) |
|  | Urban large | 29.3 (29.1–29.5) | 38.9 (38.3–39.5) |
| Pregnancy | Yes | 2.5 (2.5–2.6) | 2.6 (2.4–2.8) |
| Chronic morbidities^b^ | 0 | 74.6 (74.4–74.8) | 46.1 (45.5–46.7) |
|  | 1 | 23.4 (22.9–23.9) | 7.1 (7.0–7.2) |
|  | 2 | 7.1 (7.0–7.2) | 16.0 (15.6–16.5) |
|  | ≥3 | 5 (4.9–5.2) | 14.5 (14.1–15.0) |
| Renal disease^b^ | Yes | 0.04 (0.03–0.05) | 0.3 (0.3–0.4) |
| Osteoporosis^b^ | Yes | 1.2 (1.2–1.3) | 7.7 (7.4–8.0) |
| Epilepsy^b^ | Yes | 0.2 (0.2–0.2) | 0.5 (0.4–0.5) |
| Hyperparathyroidism^b^ | Yes | 0.02 (0.02–0.03) | 0.2 (0.2–0.3) |
| HIV^b^ | Yes | 0.09 (0.07–0.10) | 0.48 (0.39–0.56) |
| Glucocorticoids^b^ | Yes | 2.03 (1.97–2.10) | 5.62 (5.35–5.89) |
| Vitamin D supplementation^b^ | Yes | 3.2 (3.1–3.3) | 24.0 (23.5–24.5) |
| *CHF* Swiss francs, *GP* General practitioner, *HMO* Health Maintenance Organization, *HIV* Human Immunodeficiency Virus  ^a^based on definitions of Federal Statistical Office  ^b^based on pharmaceutical claims | | | |
