# Supplementary material for: Potentially inappropriate testing for vitamin D deficiency: a cross-sectional study in Switzerland
Source: BMC Health Serv Res. 2020 Nov 27;20:1097. doi: 10.1186/s12913-020-05956-2 (PMC7694269; doi:10.1186/s12913-020-05956-2)
Supplement: Supplementary file 4 — Additional file 4: Table S4. Alternative regression results: Age in 5-year groups. [file 12913_2020_5956_MOESM4_ESM.docx]

**Table S4 Online Appendix. Alternative regression results: Age in 5-year groups**

| Variable |  | Odds Ratio (95% confidence interval) |
| --- | --- | --- |
| Gender | Female | 1 |
|  | Male | 0.56 (0.13-0.15) |
| Age group in years | 19–25 | 1 |
|  | 26-30 | 1.1 (1.03-1.18) |
|  | 31-35 | 1.27 (1.18-1.36) |
|  | 36-40 | 1.43 (1.34-1.53) |
|  | 41-45 | 1.46 (1.36-1.56) |
|  | 46-50 | 1.56 (1.46-1.66) |
|  | 51-55 | 1.61 (1.51-1.71) |
|  | 56-60 | 1.55 (1.45-1.65) |
|  | 61-65 | 1.54 (1.45-1.65) |
|  | 66-70 | 1.4 (1.31-1.5) |
|  | 71-75 | 1.37 (1.28-1.47) |
|  | 76-80 | 1.37 (1.27-1.47) |
|  | 81-85 | 1.04 (0.96-1.13) |
|  | 86-90 | 0.9 (0.82-0.99) |
|  | 91+ | 0.54 (0.48-0.62) |
| Deductible class in CHF | 300 | 1 |
|  | 500 | 0.94 (0.9-0.97) |
|  | 1000 | 0.7 (0.66-0.75) |
|  | 1500 | 0.57 (0.54-0.6) |
|  | 2000 | 0.51 (0.47-0.54) |
|  | 2500 | 0.46 (0.44-0.47) |
| Health plan | Unrestricted access | 1 |
|  | Gatekeeping by GP | 1.01 (0.97-1.05) |
|  | Gatekeeping by telemedical provider | 1.04 (1-1.08) |
|  | HMO | 0.85 (0.82-0.88) |
| Supplementary insurance coverage | No | 1 |
|  | Yes | 1.21 (1.17-1.24) |
| Geographic region | Eastern | 1 |
|  | Central | 1.04 (0.98-1.09) |
|  | Mittelland | 1.07 (1.02-1.11) |
|  | Northwestern | 1.01 (0.96-1.06) |
|  | Western | 1.13 (1.08-1.19) |
|  | Zurich | 1 (0.95-1.05) |
| Urbanity levels^a^ | Rural | 1 |
|  | Peri-urban | 1.11 (1.06-1.16) |
|  | Urban small | 1.12 (1.05-1.18) |
|  | Urban midsize | 1.22 (1.16-1.28) |
|  | Urban large | 1.39 (1.33-1.46) |
| Pregnancy | No | 1 |
|  | Yes | 1.81 (1.69-1.95) |
| Chronic morbdities^b^ | 0 | 1 |
|  | 1 | 1.82 (1.76-1.88) |
|  | 2 | 2.08 (2-2.17) |
|  | ≥3 | 2.36 (2.25-2.47) |
| Renal disease^b^ | No | 1 |
|  | Yes | 1.9 (1.29-2.8) |
| Osteoporosis^b^ | No | 1 |
|  | Yes | 2.15 (2-2.3) |
| Epilepsy^b^ | No | 1 |
|  | Yes | 0.91 (0.73-1.13) |
| Hyperparathyroidism^b^ | No | 1 |
|  | Yes | 2.79 (1.64-4.73) |
| HIV^b^ | No | 1 |
|  | Yes | 1.63 (1.31-2.03) |
| Glucocorticoids^b^ | No | 1 |
|  | Yes | 1.42 (1.33-1.52) |
| Vitamin D supplementation^b^ | No | 1 |
|  | Yes | 3.91 (3.76-4.06) |

*CHF* Swiss francs, *GP* General practitioner model, *HMO* Health Maintenance Organization, *HIV* Human Immunodeficiency Virus

^a^based on definitions of Federal Statistical Office

^b^based on pharmaceutical claims
