# Supplementary material for: Potentially inappropriate testing for vitamin D deficiency: a cross-sectional study in Switzerland
Source: BMC Health Serv Res. 2020 Nov 27;20:1097. doi: 10.1186/s12913-020-05956-2 (PMC7694269; doi:10.1186/s12913-020-05956-2)
Supplement: Supplementary file 6 — Additional file 6: Table S6. Alternative regression results: Estimated average marginal effects of 10-year increase in age. [file 12913_2020_5956_MOESM6_ESM.docx]

**Table S6 Alternative regression results: Estimated average marginal effects of 10-year increase in age**

| Age | Marginal Effect of a ten-year increase (95% confidence interval) |
| --- | --- |
| 20 | 0.03 (0.02 – 0.03) |
| 30 | 0.02 (0.01 – 0.03) |
| 40 | 0.01 (0 – 0.02) |
| 50 | 0 (-0.01 – 0.01) |
| 60 | -0.02 (-0.03 – -0.01) |
| 70 | -0.04 (-0.05 – -0.03) |
| 80 | -0.06 (-0.07 – -0.05) |
