# Supplementary material for: Potentially inappropriate testing for vitamin D deficiency: a cross-sectional study in Switzerland
Source: BMC Health Serv Res. 2020 Nov 27;20:1097. doi: 10.1186/s12913-020-05956-2 (PMC7694269; doi:10.1186/s12913-020-05956-2)
Supplement: Supplementary file 7 — Additional file 7: Table S7. Association of characteristics and testing for vitamin D level in 2015 (multivariable regression analysis). [file 12913_2020_5956_MOESM7_ESM.docx]

**Table S7 Online Appendix. Association of characteristics and testing for vitamin D level in 2015 (multivariable regression analysis)**

| Variable |  | Odds Ratio (95% confidence interval) |
| --- | --- | --- |
| Gender | Female | 1 |
|  | Male | 0.53 (0.52–0.55) |
| Age group in years | 19–40 | 1 |
|  | 41–60 | 1.45 (1.40–1.51) |
|  | 61–80 | 1.41 (1.35–1.48) |
|  | 81+ | 0.93 (0.87–0.99) |
| Deductible class in CHF | 300 | 1 |
|  | 500 | 0.98 (0.94–1.01) |
|  | 1000 | 0.70 (0.66–0.74) |
|  | 1500 | 0.61 (0.58–0.64) |
|  | 2000 | 0.48 (0.44–0.52) |
|  | 2500 | 0.46 (0.43–0.49) |
| Health plan | Unrestricted access | 1 |
|  | Gatekeeping by GP | 1.06 (1.01–1.10) |
|  | Gatekeeping by telemedical provider | 1.06 (1.01–1.10) |
|  | HMO | 0.91 (0.86–0.95) |
| Supplementary insurance coverage | No | 1 |
|  | Yes | 1.22 (1.18–1.26) |
| Geographic region | Eastern | 1 |
|  | Central | 1.15 (1.08–1.23) |
|  | Mittelland | 1.17 (1.11–1.23) |
|  | Northwestern | 1.09 (1.02–1.15) |
|  | Western | 1.21 (1.15–1.28) |
|  | Zurich | 1.07 (1.00–1.13) |
| Urbanity levels^a^ | Rural | 1 |
|  | Peri-urban | 1.15 (1.06–1.22) |
|  | Urban small | 1.04 (0.97–1.11) |
|  | Urban midsize | 1.17 (1.11–1.24) |
|  | Urban large | 1.55 (1.47–1.64) |
| Pregnancy | No | 1 |
|  | Yes | 1.52 (1.40–1.66) |
| Chronic morbdities^b^ | 0 | 1 |
|  | 1 | 1.89 (1.82–1.97) |
|  | 2 | 2.13 (2.03–2.23) |
|  | ≥3 | 2.25 (2.14–2.37) |
| Renal disease^b^ | No | 1 |
|  | Yes | 1.39 (0.95–2.03) |
| Osteoporosis^b^ | No | 1 |
|  | Yes | 1.96 (1.83–2.11) |
| Epilepsy^b^ | No | 1 |
|  | Yes | 1.15 (0.92–1.45) |
| Hyperparathyroidism^b^ | No | 1 |
|  | Yes | 2.28 (1.41–3.70) |
| HIV^b^ | No | 1 |
|  | Yes | 3.54 (2.76–4.55) |
| Glucocorticoids^b^ | No | 1 |
|  | Yes | 1.26 (1.17–1.35) |
| Vitamin D supplementation^b^ | No | 1 |
|  | Yes | 4.66 (4.46–4.87) |

*CHF* Swiss francs, *GP* General practitioner model, *HMO* Health Maintenance Organization, *HIV* Human Immunodeficiency Virus

^a^based on definitions of Federal Statistical Office

^b^based on pharmaceutical claims
